# Effects of substituting commercial concentrate with chaya forage meal on feed efficiency and weight gain of growing goats fed fresh leucaena-based diets

**DOI:** 10.1093/tas/txag095

**Published:** 2026-07-07

**Authors:** Marjuki Marjuki, Asri N Huda, Tri E Susilorini, Kuswati Kuswati, Yacobus Christoper, Ahmad H Husnassabil, Chalermpon Yuangklang

**Affiliations:** Department of Animal Nutrition, Faculty of Animal Science and Technology, Universitas Brawijaya, Malang 65145, Indonesia; Department of Animal Nutrition, Faculty of Animal Science and Technology, Universitas Brawijaya, Malang 65145, Indonesia; Department of Animal Production, Faculty of Animal Science and Technology, Universitas Brawijaya, Malang 65145, Indonesia; Department of Animal Production, Faculty of Animal Science and Technology, Universitas Brawijaya, Malang 65145, Indonesia; Department of Animal Nutrition, Faculty of Animal Science and Technology, Universitas Brawijaya, Malang 65145, Indonesia; Department of Animal Nutrition, Faculty of Animal Science and Technology, Universitas Brawijaya, Malang 65145, Indonesia; Department of Animal Nutrition, Faculty of Animal Science and Technology, Universitas Brawijaya, Malang 65145, Indonesia; Department of Animal Science, Faculty of Agricultural Innovation and Technology, Rajamangala University of Technology Isan, Nakhon Ratchasima, 30000, Thailand

**Keywords:** chaya, *Cnidoscolus aconitifolius*, green concentrate, leucaena, sustainable feeding

## Abstract

This study evaluated the effect of the use of substituted concentrate with chaya (*Cnidoscolus aconitifolius*) forage meal in leucaena (*Leucaena leucocephala*) fresh forage-based diet on feed efficiency and body weight gain of growing female goats in ten weeks data collection period. Chaya forage meal was prepared from the leaves and young stems where the leaves are present. Twenty growing female etawa crossbred goats of 20.84 ± 2.48 kg initial body weight were assigned to four dietary treatments in a randomized block design of 5 blocks. The treatment diets were P0 = 100% fresh leucaena forage (Negative control); P1 = 50% fresh leucaena forage + 50% concentrate (Positive control); P2 = 50% fresh leucaena forage + 50% mixed concentrate (40% concentrate + 10% chaya forage meal); and P3 = 50% fresh leucaena forage + 50% mixed concentrate (30% concentrate + 20% chaya forage meal). The treatment diets were fed to the goats at level of 5% body weight in dry matter basis. The results showed that substitution of 50% of leucaena fresh forage as a single diet (P0) with concentrate that were either not substituted (P1) or partially substituted with chaya forage meal (P2 and P3) decreased very significantly (*P* < 0.01) total dry matter (*DM*), organic matter (*OM*), crude protein (*CP*), extract ether (*EE*), and crude fibre (*CF*) intake, but increased very significantly (*P* < 0.01) nitrogen free extract (*NFE*) intake. In addition, the DM ratio of leucaena: concentrate in the consumed diet P1-P3 treatments diet (51.67:48.33%) was much better compared to those in the P0 treatment diet (100:0). Consequently, P1-P3 treatment diets showed very significantly higher average daily weight gain (*ADG*) and very significantly higher feed efficiency (*P* < 0.01). The highest body weight gain and the highest feed efficiency were observed in P2 treatment diet. It is concluded that substitution of 50% of leucaena fresh forage as a single diet, especially with mixed of 40% concentrate and 10% chaya forage meal increased the efficiency of feed utilization and growth, offering more effective in utilizing locally feed resources and thus more sustainable feeding strategy for smallholder goat production.

## Teaser

Replacing part of commercial concentrate goat feed with 20% chaya leaf meal helped goats to grow faster while consuming less feed and utilizing the feed more efficiently. This simple and effective utilization of locally available feed must reduce feeding expenses and improve the sustainability of smallholder goat farming.

## Introduction

Goat production plays an important role in the livelihoods of smallholder farmers in tropical and subtropical regions, providing meat, milk, and income security (Devendra 2001; Escareño et al. 2012; Woldu et al. 2016; Mataveia et al. 2021; Ouchene-Khelifi et al. 2021). However, productivity is often constrained by the feeding systems. Smallholder goat farmers typically feed their goats a single, locally available tree/shrub forage collected from the plant shadow of coffee crops, *Leucaena leucocephala* or *Calliandra calothyrsus*. Feeding a single type of feed tends to cause an imbalanced nutrient in diet. Several farmers also use commercial concentrate feed as a supplement to the forage, especially during the dry season when the production of such forage is low. The concentrate feed is formulated to supply essential nutrients, particularly energy and protein that may be deficient in forage-based diets. However, concentrate feed is costly for most farmers, which limits their access to this feed. In many rural systems, feed costs can represent more than 60% of total production expenses, prompting the need for cost-effective, locally available feed resources to maintain production while improving profitability and sustainability (Molina-Alcaide et al. 2010; Lukuyu et al. 2011; Ho et al. 2018).

Chaya (*Cnidoscolus aconitifolius*) is a perennial shrub native to Central America, now cultivated in various tropical regions due to its adaptability, high biomass yield, and minimal agronomic requirements (Ross-Ibarra and Molina-Cruz 2002; Victor et al. 2016). This shrub is widely grown in Indonesia as a live fence and ornament on the roadside. These plants are deep-rooted and have dense leaves that survive during the dry season. The leaves of this shrub are rich in crude protein ranging from 14.7% (Shittu et al. 2014) to 34.2% (Kongphapa et al. 2022), minerals such as calcium, potassium, and iron, and vitamins, including ascorbic acid and β-carotene (Donkoh et al. 1999; Aguilar-Ramírez et al. 2000; Shittu et al. 2014). Higher protein content of 32.41–34.02g/100g leaves dry weight was reported by Kongphapa et al. (2021) and Lennox and John (2018). Additionally, chaya contains bioactive compounds with antioxidant and antimicrobial properties (Lennox and John 2018). which may offer secondary benefits in animal health. While the plant contains cyanogenic glycosides, these are effectively reduced through wilting or cooking before feeding (Kuti and Konoru 2006; Oduguwa et al. 2006; John and Opeyemi 2015).

Several studies have reported the potential of chaya as a feed supplement for poultry (Aguilar-Ramírez et al. 2000; Kese, Donkoh, Atuahene, and Nkansah 2021; Kese, Donkoh, Atuahene, and Poku-Prempeh 2021; Wongnhor et al. 2023), rabbits (Lawal et al. 2010), and ruminants (Kumar et al. 2010, Kumar et al. 2011; Totakul et al. 2021), blue shrimp (Rocha Estrada et al. 2012). Poot‐López and Gasca‐Leyva (2009) reported that chaya leaves can be used to substitute a balanced feed in tilapia diet up to 50% to get the lowest feed cost, but no significant difference in weight gain was observed with those in the control diet of 100% balanced feed. In ruminant nutrition, the high protein content and year-round availability of chaya forage make it a promising feed for age to balance low-quality basal diets, such as crop residues or native grasses. However, there is limited empirical evidence on its role as a direct substitute for commercial concentrate feeds in goats. Such substitution could reduce feed costs, enhance self-sufficiency, and contribute to more resilient ruminant feeding systems for smallholders.

Understanding the implications of replacing concentrates with chaya forage requires a comprehensive evaluation of intake, nutrient digestibility, growth performance, and economic returns in goat production. Therefore, this study aimed to determine the effects of partially substituting commercial concentrate with chaya forage in the diet of growing female goats.

## Materials and methods

### Experimental animals

A total of 20 growing female Etawah crossbred goats, with an initial average body weight of 20.84 ± 2.48 kg, were used in this study. All animals were clinically healthy and had been acclimated to their housing and diets for fourteen days before the start of data collection. The goats were housed individually in well-ventilated pens equipped with feeding troughs and water containers to allow accurate measurement of feed intake. All animal procedures were approved by the Research Ethics Committee (Animal Care and Use Committee), Faculty of Veterinary Medicine, Universitas Brawijaya, Malang, East Java Province, Indonesia No. 99-KEP-FKHUB-2026.

### Experimental design and diets

The experiment used a randomized block design (RBD) with four dietary treatments and five replicates or blocks per treatment. Blocking of the goats was based on the initial body weight of each goat. The treatments were four different experimental diets consisting of fresh *L. leucocephala* forage as the basal feed, supplemented with concentrates that were either not substituted or substituted with chaya forage meal as below.


P0=100% fresh leucaena forage (Negative control)

P1=50% fresh leucaena forage+50% concentrate (Positive control)

P2=50% fresh leucaena forage+50% mixed concentrate(40% concentrate+10% chaya forage meal)

P3=50% fresh leucaena forage+50% mixed concentrate(30% concentrate+20% chaya forage meal)

The composition of feed ingredients in each treatment diets (% in DM weight basis) is presented in Table 1.

**Table 1:** Composition of feed ingredients in each treatment diets (% in DM weight basis).

Treatments	Fresh Leucaena Forage	Concentrate	Chaya Forage Meal
**P0**	100	0	0
**P1**	50	50	0
**P2**	50	40	10
**P3**	50	30	20

*Notes*.

-Concentrate and chaya forage meal in P2 and P3 were thoroughly mixed.

-The total daily feed allowance in each treatment was fixed at 5.0% of body weight in DM basis.

The treatment diets were offered twice daily at 08:00 and 16:00 h, with clean drinking water provided ad libitum. The total daily feed offered was set in DM basis at 5.0% of each goat body weight. This setting was higher than the average DM intake by goats as reported by (de Almeida et al. 2019; Ruvuga et al. 2022; Nipane et al. 2023) that was 3% of body weight to give allowance to the goats in consuming the treatment diets.

Fresh *L. leucocephala* forage was harvested daily from coffee plantation areas near the site of the experiment. Chaya forage meal was prepared from leaves and young stems where the leaves are present. The harvested chaya forage was sun-dried to reach approximately 85% to 90% dry matter content, and then ground through a 1-mm sieve. The commercial concentrate feed was bought from the feed mill of “Jabung” dairy cooperative in Jabung subdistrict, Malang Regency. The concentrate was composed of main ingredients including wheat pollard, rice bran, molasses, corn gluten feed (CGF), dried distillers’ grain with soluble (DDGS), copra meal, mix-minerals, however, the composition was not mentioned by the feed mill. Nutrients content of each feed ingredient used in the treatment diets is presented in Table 2.

**Table 2:** Nutrients content of feed ingredients used in each treatment diets of this experiment.

Sample	Nutrient contents						
	DM (%)	Ash (% DM)	OM (% DM)	CP (% DM)	EE (% DM)	CF (% DM)	NFE (% DM)
***L. leucocephala* forage**	28.27	7.87	92.13	25.26	3.02	33.67	30.18
**Concentrate feed**	85.39	8.37	91.63	17.38	5.85	8.22	60.18
**Chaya forage meal**	91.70	10.03	89.97	11.44	4.94	31.31	42.28
**Concentrate in P1**	85.39	8.37	91.63	17.38	5.85	8.22	60.18
**Concentrate in P2**	86.65	8.70	91.30	16.19	5.67	12.84	56.60
**Concentrate in P3**	87.91	9.03	90.97	15.00	5.49	17.46	53.02
**Treatment diets:**							
**P0**	28.27	7.87	92.13	25.26	3.02	33.67	30.18
**P1**	56.83	8.12	91.88	21.32	4.44	20.95	45.18
**P2**	57.46	8.29	91.71	20.73	4.34	23.25	43.39
**P3**	58.09	8.45	91.55	20.13	4.25	25.56	41.60

DM, dry matter, OM, organic matter, CP, crude protein, EE, extract ether, CF, crude fibre, NFE, nitrogen free extract.

Fresh leucaena forage and concentrate were given to goats at the same time but in separately places. Leucaena forage was given by tying it into a bundle and then hanging it above the feeding trough. The concentrate was given in a plastic bucket which was put in the feeding trough.

### Data collection

Data collection was done for ten weeks period, following after two weeks adaptation period and two weeks preliminary period, respectively. The collected data were feed intake, body weight gain and feed efficiency.

#### Feed intake

Feed intake was measured for twenty-four hours period every two days during the data collection period. Feed was offered and refusals were weighed. Samples of *L. leucocephala* forage offered to all goats and refusals of individual goat were collected, immediately chopped, and sun-dried to prevent the samples from spoiling. Samples of concentrate feed in the treatment P1 to P3 were periodically collected every week. After finishing data collection period, all samples of feed offered were composited according to feed ingredient, while all samples of refusal feed were composited according to feed ingredient and individual goat. Sub-samples of each composited samples were taken for laboratory analysis of their nutrient contents.

#### Body weight gain and feed efficiency

Each goat was weighed at the start of the trial and every two weeks thereafter, before the morning feeding. ADG was measured every two weeks and calculated as the difference between final and the initial body weight of two consecutives weighing time, divided by 14 days and then averaged for the whole measurements. Feed efficiency was calculated as the ratio of average daily gain (ADG, g/head/day) to total daily dry matter intake (DMI, g/head/day). Feed efficiency = ADG/DMI. Higher feed efficiency values indicate a better feed utilization by the goats.

#### Chemical analysis

Samples of feed offered and refusal feeds were ground and analysed for DM, ash, OM, CP, EE, CF and calculated NFE content according to AOAC (2005) procedures. The laboratory analysis was done in the Laboratory of Animal Feed and Nutrition, Faculty of Animal Science and Technology, Universitas Brawijaya in Malang, East Java Province, Indonesia.

#### Statistical analysis

Data on feed intake, ADG, and feed efficiency were subjected to one-way analysis of variance (ANOVA) of randomized block design using statistical software SAS 9.4. Significant treatment differences (*p* < 0.05) were evaluated using Duncan’s Multiple Range Test (DMRT).

## Results and discussion

Nutrient contents, intake, and digestibility are key parameters in the evaluation of the potential of feedstuffs in supplying nutrients required by livestock for maintenance and production. The parameters determine directly the nutritive, biological, and economical value of the feed for the livestock production, as well as its environmental impact (Coleman and Moore 2003), thereby influencing the overall sustainability of the livestock production system, which is a primary goal in modern livestock production.

### Nutrients content

Nutrients content of feed ingredients used in the treatment diets evaluated in this experiment is presented in Table 2. CP content of leucaena forage (25.26%) in the present study was in the range of reported by (Lani et al. 2015; Santos et al. 2017; Manuel et al. 2020; Simbaya et al. 2020; Verdecia et al. 2020; Zapata-Campos et al. 2020). The lowest CP content leucaena leaves was 19.8% (Lani et al. 2015; Navale et al. 2022) and the highest was 33.2% (Verdecia et al. 2020; Katoch et al. 2026). The variation in CP content of leucaena is mostly affected by cutting age or interval and season of the year. Longer cutting interval or older age of leucaena increased fibre content, but decreased consistently CP content and digestibility (Kant et al. 2017; Simbaya, Chibinga, and Salem 2020; Sulistijo et al. 2020; Verdecia et al. 2020; Navale et al. 2022; Castro-González et al. 2025; Katoch et al. 2026). However, seasons differentially affect the nutrient content of leucaena between tropical and temperate regions. In tropical areas, the CP content of leucaena is higher during the rainy season than the dry season (Sulistijo et al. 2020; Verdecia et al. 2020), which is attributed to greater soil nutrient availability. In contrast, in temperate regions, the CP content peaks during spring and summer compared to autumn and winter, driven by increased sunlight and more intensive photosynthesis (Navale et al. 2022; Castro-González et al. 2025; Katoch et al. 2026).

The CP of chaya forage meal used in the present study (11.44% DM) was lower than values reported in previous studies, which ranged from 14.7% (Shittu et al. 2014) to 34.2% (Kongphapa et al. 2022). The discrepancy is likely attributable to differences in sample composition. In the present study, chaya forage meal consisted of both leaves and young stems, whereas many published studies analysed leaf samples only as vegetables for human consumption (Kuti and Konoru 2006; Ebel et al. 2019; Pérez-González et al. 2021; Ramírez Rodrigues et al. 2021), as non-ruminant feed components (Donkoh et al. 1999; Kese, Donkoh, Atuahene, and Nkansah 2021; Kese, Donkoh, Atuahene, and Poku-Prempeh 2021; Wongnhor et al. 2023), or as fish or shrimp feed components (Poot‐López and Gasca‐Leyva 2009; Rocha Estrada et al. 2012; Khieokhajonkhet et al. 2024). Since stems generally contain lower crude protein and higher structural carbohydrate concentrations than leaves, their inclusion would be expected to reduce the overall crude protein concentration of the forage meal. Similar variations in nutrient composition associated with plant fraction and maturity have been reported for numerous tropical shrub forages.

Importantly, the use of leaves and young stems in the present study may better represent actual feeding practices of smallholder farmers, who generally harvest and utilize whole edible portions of the plant rather than separating leaves from stems. Therefore, the nutrient composition reported herein may provide a more realistic estimate of the feeding value of chaya forage under field conditions.

Data in Table 2 showed that fresh leucaena forage contained lower DM, ash, EE, and NFE, but higher in OM, CP, and CF than those of the concentrate and chaya forage meal. In addition, the concentrate contained lower DM, ash and CF, but higher in OM, CP, EE and NFE than those of chaya forage meal. Consequently, the nutrient content of P0 treatment diet that was composed only fresh leucaena forage was lower in ash, EE and NFE but higher OM, CP and CF than those of P1, P2, and P3 diets, that were composed 50% fresh leucaena forage and 50% concentrate, which was either not substituted or partially substituted with chaya forage meal.

Based on the nutrients content of each treatment diets in Table 2, P0 treatment diet which was composed of only fresh leucaena forage showed imbalance nutrient content. The P0 treatment diet contained much higher CP (25.26% DM) in respect of the requirement by goats, but contained lower energy as expressed by the low NFE content. P1 treatment diet, which was composed of 50% fresh leucaena forage and 50% concentrate, as well as, P2 and P3 treatment diets, which were composed 50% fresh leucaena forage and 50% concentrate containing chaya forage meal, must have better balance nutrient content and have better associative effect between the feed ingredients than P0 treatment diet.

### Feed intake

DM intake is the most common measure of feed evaluation and a critical parameter for nutrients intake. This is because of all nutrients, except water, are contained within the dry matter fraction (Gurgel et al. 2021; Uskenov et al. 2024). Data on DM intake of each feed ingredient and total nutrient intake by growing female goat fed on only fresh leucaena forage diet (P0), 50% fresh leucaena forage and 50% concentrate feed which were either not substituted (P1) or partially substituted with chaya forage meal (P2 and P3), body weight gain, and feed efficiency are presented in Table 3.

**Table 3:** Feed intake, body weight gain, and feed efficiency of growing female goats fed on leucaena forage based diet and concentrate feed, either not substituted or substituted with chaya forage meal.

Variables	Treatment				SEM	*p*-Value	Post-hoc	Note
	P0	P1	P2	P3			Power (%)	
**DM Intake**								
***Leucaena forage***								
**DM intake (g/head/day)**	813.69	401.38	404.55	407.97	10.522	0.908	6.31	*
**DM intake (% BW)**	3.44	1.62^a^	1.63^a^	1.68^b^	0.009	0.004	99.52	*
**DM Refusal (% BW)**	1.56	0.88^b^	0.87^b^	0.82^a^	0.009	0.004	99.52	*
***Concentrate***								
**DM intake (g/head/day)**	0	378.87	372.55	376.36	5.082	0.688	10.62	*
**DM intake (% BW)**	0	1.54	1.51	1.56	0.024	0.371	23.07	*
**DM Refusal (% BW)**	0	0.96	0.99	0.94	0.024	0.371	23.07	*
***Total***								
**DM intake (g/head/day)**	813.69	780.25	777.10	784.32	15.058	0.336	25.32	*
**DM intake (% BW)**	3.44^b^	3.16^a^	3.14^a^	3.24^a^	0.032	0.000	99.99	*
**DM Refusal (% BW)**	1.56^a^	1.84^b^	1.86^b^	1.76^b^	0.032	0.000	99.99	*
**Leucaena : Concentrate DM intake ratio**	100:0	51:49	52:48	52:48	–	–		na
**Total Nutrient Intake (g/kg BW^0.75^/day)**								
**DM**	75.84^b^	70.27^a^	69.92^a^	71.72^a^	0.857	0.001	98.23	–
**OM**	69.80^b^	64.87^a^	64.37^a^	65.88^a^	0.826	0.002	96.73	–
**CP**	20.21^b^	15.58^a^	15.10^a^	15.35^a^	0.193	0.000	100.00	–
**EE**	2.47^c^	3.38^d^	2.07^b^	1.92^a^	0.044	0.000	100.00	–
**CF**	25.41^b^	13.96^a^	13.95^a^	14.53^a^	0.323	0.000	100.00	–
**NFE**	21.71^a^	31.94^b^	33.26^c^	34.08^c^	0.316	0.000	100.00	–
**Body Weight, Gain, and Feed Efficiency**								
**Initial body weight (kg)**	20.84	20.82	20.84	20.84	–	–		na
**Final body weight (kg)**	25.38	27.23	28.70	26.20	–	–		na
**Weight gain (g/head/day)**	64.80^a^	91.63^c^	112.24^d^	76.63^b^	1.776	0.000	100.00	–
**Feed Efficiency**	0.082^a^	0.121^c^	0.146^d^	0.99^b^	0.003	0.000	100.00	–

DM, dry matter, OM, organic matter, CP, crude protein, EE, extract ether, CF, crude fibre, NFE, nitrogen free extract, g, gram, kg, kilogram, BW, Body weight, Feed Efficiency, Weight gain/Total DM intake.

* = only P1, P2, and P3 are subjected to Anova, P0 was not included in the Anova because of as a strictly different treatment (Negative control), na, Not subjected to Anova.

Data in Table 3 showed that the goats fed on each treatment diet exhibited DM intake in the range of 3.14% to 3.44% BW. These data were in more or less same range as those reported by (Nipane et al. 2023) and (Wandara et al. 2020) where the daily feed DM intake of goats ranged from 1.9% to 3.8% and from 3.29% to 3.67% of body weight, respectively. However, the DM intake data measured in this experiment were slightly higher than those reported by de Almeida et al. (2019)  and Ruvuga et al. (2022). de Almeida et al. (2019) summarized DM intake data of 515 goats from four studies conducted in Brazil at an altitude of 595 m. The goats were fed ad libitum similar diets composed of dehydrated corn plant (*Zea mays*; 46.2 ± 0.7%) or Tifton hay (*Cynodon dactylon*; 24.5 ± 0.3%), corn grain (33.4 ± 9.2%), soybean meal (17.6 ± 2.9%), soybean oil (1.3 ± 0.5%), limestone (1.1 ± 0.3%), mineral supplement (2.2 ± 0.9%), and ammonium chloride (0.7 ± 0.4%). The formulated diets had 16.2 ± 2.01% CP and 2.65 ± 0.332 Mcal of ME/kg DM. Their results showed that the average DM intake of the goats was 1.9 to 4.1% of body weight, with an average of 3.01% of body weight. Ruvuga et al. (2022) also reported that the average DM intake of growing Tanzanian dual-purpose Blended goats (55% Kamorai, 30% Boer, 15% Small East African goat) was 3.08% of body weight.

The use of concentrate, which was either substituted or not substituted with chaya forage meal in leucaena forage-based did not significantly affect total DM intake (*p* > 0.05). So, in this case, the goats did a substitutional DM intake of fresh leucaena forage with concentrate rather than an additional intake. The results were in line with those of Tedeschi et al. (2019), but in contrast to the data reported by (de Lima Júnior et al. 2025). Tedeschi et al. (2019) reported that when a feed supplement is included in the diet of a ruminant fed pasture only, then the total DM intake can vary unexpectedly. This phenomenon constitutes the substitution effect, which is measured as the substitution rate (SR). The substitution rate (SR) is the ratio of the difference between herbage intake in the un-supplemented and supplemented feed to the intake of the feed supplement itself. The SR value can be positive or negative. A positive SR value means that intake of feed supplement is associated with a reduction in herbage intake. A positive SR value generally occurs when DM intake of un-supplemented herbage is already high or has fulfilled the animal’s intake requirement although without feed supplement. This mostly occurs when the animal is fed high-quality herbage in sufficient quantity. In contrary, a negative SR value means that intake of feed supplement is followed by an increase in herbage intake. A negative SR value generally occurs when DM intake of un-supplemented herbage is lower than the animal’s intake requirement. This condition mostly occurs when the animal is fed a sufficient quantity of herbage but low in quality. A negative SR value was reported by (de Lima Júnior et al. 2025), when grazing goats in dry tropical agroforestry (Caatinga) were supplemented with concentrate at four different levels (0, 0.5, 1.0, and 1.5% of body weight), the increase in the level of concentrate supplementation increased concentrate and forage intake, which finally increased total DM intake.

The average SR value of fresh leucaena forage and concentrate of either not substituted or substituted with chaya forage meal in this present study was 1.09 ± 0.01. This was because of the total DM intake of fed alone fresh leucaena forage (P0 treatment diet) was already high, 3.44 ± 0.29% body weight.

Total DM intake as expressed in g/kg BW^0.75^/day in this experiment ranged from 69.92 to 75.84. Closely similar data were reported by (Dutta et al. 2025), from 68.53 to 89.83 with SEM 2.92, in growing goats receiving ad libitum bengal gram straw and green fodder as basal feed, supplemented with barley grain at 0.7% of body weight in the control group, and concentrate mix at 0.7%, 1.4%, and 2.1% of body weight for treatment groups, respectively. While, slightly lower data were reported by (Wandara et al. 2020), from 61.69 to 66.66, in woyto-guji goats that were fed on ad libitum grass hay with with feed supplement of substituted concentrate with dried moringa (*Moringa stenopetala*) leaves at the rate of 1.5% body weight. A higher data were reported by (Olafadehan et al. 2020), from 83.61 to 92.15, in goats with free access to threshed sorghum tops and 50 g/kg of their LW on DM basis of concentrate partially (25 and 50%) replaced with daniellia foliage.

The feed intake parameters of growing female goats subjected to the experimental diets in this present study showed that daily total DM intake expressed in absolute volume (g/head/day) did not vary significantly among treatments (*p* = 0.336), ranging from 777.10 g/head/day in P2 to 813.69 g/head/day in P0. However, when the DM intake data were normalized against body weight (% BW), then total DM intake demonstrated highly significant differences (*p* = 0.000) among the treatments. Goats in the 100% fresh leucaena forage group (P0) exhibited the highest intake (3.44% BW), while supplemented feed treatments with concentrate either not substituted or substituted with chaya forage meal (P1, P2, and P3) showed an explicit drop down to 3.16% BW to 3.14% BW, and 3.24% BW, respectively.

The higher DM intake relative to body weight observed in the sole leucaena forage group (P0) indicates a compensatory consumption mechanism. Ruminants typically increase volumetric consumption of fibrous, low-energy forages to fulfil metabolic energy prerequisites up to the physical limitations of the rumen capacity. Conversely, substituting a portion of the leucaena forage with concentrate either not substituted or substituted with chaya forage meal (P1–P3) introduced highly fermentable carbohydrates, satisfying chemostatic satiety sensors faster. This mechanism limits excessive feed intake without compromising the physiological energy requirements of the animal. Furthermore, DM refusal (% BW) was significantly higher in supplemented groups than in P0 (*p* = 0.000), reinforcing that the higher nutrient density in the mixed concentrate diets met biological demands at lower relative ingestion rates.

Total nutrient consumption, evaluated on a metabolic weight basis (g/kg BW^0.75^/day), was significantly altered by the structural composition of the treatments (*p* < 0.05). The crude protein (CP) intake dropped significantly from 20.21 g in P0 to a stabilized range of 15.10–15.58 g in P1–P3 (*p* = 0.000). Similarly, crude fiber (CF) intake dropped from 25.41 g (P0) to 13.95–14.53 g in the supplemented groups (*p* = 0.000). In direct contrast, nitrogen-free extract (NFE) ingestion experienced a highly significant stepwise increase from 21.71 g (P0) to 31.94 g (P1), 33.26 g (P2), and peaking at 34.08 g in P3 (*p* = 0.000).

The elevated CP and CF intake in P0 explicitly mirrors the inherent composition of pure *Leucaena leucocephala* leaves, which are rich in structural protein and fibrous cell wall components. However, high-fibre, mono-forage legume diets are often constrained by slower rumen transit times and localized cell wall resistance. The inclusion of concentrates and chaya forage meal successfully restructured the carbohydrate profile by providing highly accessible non-structural carbohydrates (as evidenced by the climbing NFE). This shifts the nitrogen-to-energy ratio in favor of rumen synchronization. It provides a steady baseline of fermentable energy necessary for the ruminal microbiota to capture non-protein nitrogen and plant peptides efficiently.

### Body weight gain and feed efficiency

Growth or body weight gain is a manifestation of the conversion of the consumed feed into body biomass. This is a result of the combination of nutrient intake, digestibility, and the efficiency of metabolism and utilization. The average daily weight gain (ADG) exhibited highly significant differences across all treatments (*p* = 0.000), ranging from 64.80 g/head/day to 112.24 g/head/day. Goats fed the P2 ration (the use of 40% concentrate and 10% chaya forage meal substitution in the leucaena forage-based diet) achieved the most robust growth rate at 112.24 g/head/day. This performance was statistically superior to P1 (91.63 g/head/day), P3 (76.63 g/head/day), and the baseline P0 group (64.80 g/head/day).

The substantial acceleration of growth from P0 to P2 demonstrates an optimal “associative effect” of multi-source feeds. While leucaena provides excellent foundational bypass protein, its absolute biological efficiency can be hampered by mimosine toxicity and structural cell wall restrictions if fed as an exclusive ration. Chaya (*Cnidoscolus aconitifolius*) leaf meal features high concentrations of mineral complexes and essential amino acids like lysine and leucine. Chaya leaf contains moderate to high lysin but low in methionine, the two most limiting amino acids in all animals (Ramírez Rodrigues et al. 2021; Kongphapa et al. 2022). Incorporating chaya at a moderate 10% inclusion level in the diet (P2) likely triggered an up-regulation in volatile fatty acid (VFA) synthesis, favouring propionate pathway generation, which translates directly into tissue accretion and increased frame development in growing kids. As mentioned before that nutrient intake in leucaena forage diet supplemented with concentrate (P1-P3) was lower than the intake in leucaena forage-only diet (P0), except for NFE intake. Thus, the higher ADG in the treatment diets P1-P3 than in the control diet P0 must be associated by a higher availability and better balance of nutrients for body tissue deposition due to higher associative effects of more of feed types in the treatment diets especially P2 and P3 treatment diets. This phenomenon was in accordance to (Santos et al. 2018).

However, increasing chaya meal to 20% in the diet to replace concentrate (P3) caused a drop in growth performance to 76.63 g/day. This depressive trend at higher levels is a classic response to anti-nutritional compounds. Raw chaya foliage contains Some anti-nutritional factors including cyanogenic glycosides, phytate, oxalate, saponin, alkaloids, and tannin. If present in higher volumes, these compounds can impose metabolic burdens on the animal’s liver for detoxification, routing available amino acids away from muscle synthesis to execute metabolic clearance. Although the anti-nutritive factors contained in the feeds can be reduced by some treatments. However, Aye (2012) reported that sun drying was not effectively decrease the anti-nutritive contents of chaya leaf as compared to boiling or cooking. Sun dried chaya leaves still contains higher tannin, phytate, oxalate, saponin, and alkaloids compared to cooked chaya leaves (3.72 vs 2.97%, 26.78 vs 12.16 mg/g, 2.03 vs 0.81 mg/g, 7.67 vs 3.24%, and 12.07 vs 5.33%, respectively). In addition, Oyewole et al. (2025) reported that sun and solar drying of chaya leaf reduced saponin, flavonoids, alkaloid, and tannin content only at rate of 4.49, 40.85, 65.88, and 82,22%, respectively.

Feed efficiency indicates the efficiency with which animals convert consumed feed into the production. Higher feed efficiency values reflect a better consumed feed utilization by the animal. Feed efficiency indicates the efficiency with which animals convert consumed feed into the production. Higher feed efficiency values reflect a better consumed feed utilization by the animal. The results of this recent study showed that feed efficiency (daily weight gain (g)/total DM intake (g)) of goats fed on each treatment ranged from 0.082 in the P0 treatment to 0.146 in the P2. The values of feed efficiency are closely similar to those reported by Wandara et al. (2020) and Hassen (2024). Wandara et al. (2020) reported that Woyto-Guji goats fed on ad libitum grass hay supplemented with substituted concentrate with dried moringa (*Moringa stenopetala*) leaves at the rate of 1.5% body weight showed feed efficiency (daily weight gain (g)/total DM intake (g)) ranging from 0.060 to 0.097, where substitution of concentrate with dried moringa in the diet increased feed efficiency. Hassen (2024) reported that Afar goats fed on ad libitum panicum grass hay as a basal diet supplemented with substituted concentrate with alfafa showed feed efficiency ranging from 0.151 to 0.55, where the increase of substitution of concentrate with alfafa in the diet decreased feed efficiency.

This recent study data showed that feed efficiency was significantly boosted by altering the dietary matrix (*p* = 0.000). The most favourable feed efficiency was captured in the P2 treatment (the use of 40% concentrate and 10% chaya forage meal substitution in the leucaena forage-based diet), yielding a value of 0.146. This represents a massive optimization compared to the P1 group (0.121) and the exceptionally inefficient profile of the P0 treatment (0.082). The poorest feed efficiency in P0, a mono-forage treatment diet, must be due the imbalance nutrients availability and the missing of associative effects among the feed ingredients in the consumed diets. It was shown in Table 3 that the DM ratio of leucaena to concentrate in the consumed diet was on average of 51.67: 48.33% in the P1-P3 treatments diet compared to those in the P0 treatment diet that was 100: 0.

Consequently, feeding sole *Leucaena* results in wasted feed biomass per unit of live-weight gain. Restructuring the ration by including concentrate and also chaya forage meal in the diet improves the nutrients balance and the associative effects in the diet. The highest feed efficiency was shown in goats fed on P2 treatment diet, indicating the highest of feed utilization efficiency. The improvement aligns with previous findings that the inclusion of high-protein green forages in concentrate mixtures can enhance nutrient utilization and growth rate in ruminants (Santos et al. 2018).

The overall results of the present study suggest that partial replacement of concentrate feed with chaya forage meal can maintain feed intake but increase nutrients degradability, and body weight gain in growing female goats. The optimal inclusion level of chaya forage meal in the concentrate was up to 20% in DM basis, which yielded the highest ADG and highest feed efficiency. Given the year-round availability and low production cost of chaya, its strategic use as a green concentrate component could enhance the sustainability and profitability of smallholder goat production systems.

## Conclusion

Partial substitution of commercial concentrate with *C. aconitifolius* (chaya) forage meal, specifically at a 20% substitution level within the concentrate portion (equivalent to 10% of the total diet), optimizes nutrient balance, increases average daily gain, and lowers the feed conversion ratio in growing goats fed a 50% fresh leucaena forage and 50% concentrate in DM basis. Chaya forage meal represents a sustainable, cost-effective, and drought-tolerant local resource capable of minimizing feed expenditures in smallholder production systems without hindering animal performance.

## List of abbreviations

DM: dry matter
OM: organic matter
CP: crude protein
EE: extract ether
CF: crude fibre
NFE: nitrogen free extract
ADG: average daily weight gain
RBD: randomized block design
